# Clinicopathological characteristics and treatment outcomes of occult breast cancer: a population-based study

**DOI:** 10.1186/s12893-022-01472-8

**Published:** 2022-04-17

**Authors:** Zijun Zhao, Ting Zhang, Yu Yao, Xin Lu

**Affiliations:** Department of Surgery, Peking Union Medical College Hospital, Chinese Academy of Medical Sciences and Peking Union Medical College, 1 Shuaifuyuan, Wangfujing, Beijing, 100730 China

**Keywords:** Occult breast cancer, Surgical treatment, Systemic therapy, Radiotherapy, Survival

## Abstract

**Background:**

Occult breast cancer (OBC) is a special type of breast cancer. Because of its rarity, clinicopathological information is still insufficient, causing a controversial condition about its treatment recommendation. Thus, we aimed to clarify major clinicopathological information, treatment strategies and prognosis of OBC based on a large population.

**Methods:**

We retrospectively collected adult female OBC population from Surveillance, Epidemiology, and End Results database. We divided the whole cohort into two groups based on surgical treatment in-breast. Descriptive analysis of 18 clinicopathological variables was conducted. Survival analysis was performed based on different clinicopathological factors. Univariate and multivariate Cox regression analysis was performed to identify potential independent predictor for prognosis of OBC.

**Results:**

1189 OBC patients were in final analysis and most of them were diagnosed as an early-stage carcinoma. Patients received breast-conserving treatment (BCT) was nearly two times of ones received mastectomy. Patients receiving radiotherapy in BCT group were significantly more than patients receiving radiotherapy in mastectomy group (61.76 vs. 50.9%, *P*  <   0.001). After a median follow-up period of 62 months, 5-year and 10-year overall survival (OS) of all subjects was 81.6% and 68.8%, respectively. No significant difference in OS and breast-cancer specific survival (BCSS) was found between mastectomy and local breast-conserving surgery. Older age and larger number of positive lymph nodes causes a worse prognosis whereas radiotherapy brought a better clinical outcome for OBC patients.

**Conclusions:**

OBC has a generally good prognosis. Less-intensive surgery does not negatively impact clinical outcomes of OBC while additional radiotherapy is totally beneficial to prolong OS and BCSS.

## Background

Occult breast cancer (OBC) is a special type of breast cancer. It is first reported and described by Halsted as “cancerous axillary glands with non-demonstrable cancer of the mamma” in 1907 [1]. The present definition of OBC is a kind of primary breast cancer diagnosed histologically by biopsy of axillary lymph nodes but without clinical or radiographic finding (mammography and ultrasonography) in the breast itself [2, 3]. The incidence of OBC among all types of breast cancer is about 0.1 to 1% [4].

Because of its rarity and lack of primary focus within the breast, the clinicopathological traits of OBC, including lymph node metastasis, immunohistochemistry [hormone status/human epidermal factor receptor-2 (HER-2) status, etc.], is still unclear [5–8]. Moreover, prognosis of OBC is still debatable. 10-year overall survival of OBC in different reports varied from 45% to nearly 70% [9].

The National Comprehensive Cancer Network (NCCN) guidelines of breast disease recommended that patients diagnosed with OBC by magnetic resonance imaging (MRI) be treated in two ways according to nodal condition: (1) for T0N1M0 disease, Breast conserving therapy (BCT) [axillary lymph node dissection (ALND) plus whole breast radiotherapy with or without nodal radiotherapy] or ALND plus mastectomy (modified radical mastectomy, MRM) with or without radiotherapy; (2) for T0N2-3M0 disease, neoadjuvant systemic therapy (chemotherapy, endocrine therapy and targeting therapy) followed by MRM [9, 10]. Up till now, research of OBC is largely derived from case reports as well as insufficient number of retrospective clinical studies with small sample size [11–13]. Shortage of comprehensive clinical research and insufficient number of subjects in various retrospective studies hurdled determination of a more general and individualized therapeutic options [11, 12].

In our study, we collected the latest edition of population-based data from the Surveillance, Epidemiology, and End Results (SEER) database. The main goal of our study is to further investigate the main clinicopathological features of OBC and acquire the most updated information of association between different types of treatment and prognosis.

## Methods

### Ethical statement

No patient informed consent is necessary because SEER database is publicly available and all data have been de-identified. We have submitted our application and obtained the approval of usage for the updated data of SEER database.

### Data source and study cohort

We conducted a retrospective population-based cohort study using SEER 18 registry research data (Nov 2020 submission). The database included data from 18 population-based cancer registries (2000–2018) and covered approximately 27.8% of US population. Data-retrieving software SEER*STAT software ver. 8.3.9.1 was used to collect finally wanted data. The inclusion criteria include “Female”; age of diagnosis from 18 to 85; year of diagnosis from 2004 through 2018; diagnosis is primary breast cancer; American Joint Committee on Cancer breast staging T is T0 and M is M0; A known number of examined lymph nodes; positive lymph node not less than one (N+). Diagnosis not by histology, male patients, unknown laterality; breast cancer staging not T0, N staging with N0 or Nx, breast cancer with distal metastasis and unknown staging were all excluded.

Clinicopathological variables include age, year of diagnosis, race, marital status, grade, number of examined lymph nodes, number of positive lymph nodes, N staging, surgery types, radiation, chemotherapy, types of systemic therapy, subtypes of breast cancer, estrogen receptor (ER), progesterone receptor (PR), and HER-2. For the reason that HER-2 status was not recorded until 2010, it is not available in patients whose diagnosis were between 2004 and 2009. Subtypes of breast cancer includes Luminal A (ER-positive and/or PR-positive, HER-2 negative), Luminal B (ER-positive and/or PR-positive, HER-2 positive), HER-2 positive (ER and PR-negative, HER-2 positive) and triple negative (ER negative, PR negative and HER-2 negative).

### Study endpoints and definition

In our study, OS served as primary outcome. It was defined as from the date of diagnosis to the date of death, no matter if the patient was dead from breast cancer or other reasons. Breast cancer-specific survival (BCSS) was secondary outcome. It was calculated from the day of diagnosis to the date of death caused by breast cancer.

### Statistical analysis

For continuous variables, Shapiro tests will be conducted to identify whether they meet the criteria of normal distribution. If so, t-test will be used. If not, Wilcoxon rank sum and signed rank tests will be conducted. For categorical variables, Chi-square test or Fisher’s exact test will be utilized to describe the difference of rate for every clinicopathological variables between two groups. Survival curves were illustrated by the Kaplan–Meier method and the difference in OS and BCSS rate in different groups was identified using log-rank test. Hazard ratio (HR) with 95% confidence intervals (CI) was calculated by Cox proportional hazard regression model to determine the factors significantly related with outcome. Clinicopathological factors with *P* value  <   0.1 in univariate analysis would be selected to be further analyzed in multivariate analysis. Significant association between selected factors and major outcomes was defined as *P* value of 0.05 or less. All the statistical analyses were two-sided and all the *P*-value less than 0.05 was set as a significance level except univariate analysis.

The above statistical analysis was conducted by R software version of 4.0.4. Main packages necessary for R program include "survival" (version 3.2-7), “survminer” (version 0.4.8), ”ggplot2” (version 3.3.3) and “ggthemes” (version 4.2.4).

## Results

### Baseline clinicopathological description of OBC cohort

A total of 1189 patients diagnosed as OBC were included in ultimate analysis from 2004 to 2018. Mean age of complete OBC cohort was 59.88 years and the range of age is from 26 through 85. White patients predominated the whole population with a percentage of 79.20% while Asian or Pacific islander accounted for nearly 10%. Majority of OBC patients were married women (n  =  699, 58.80%). Ductal carcinoma was the main histological types of OBC. For N staging and the general staging, patients with stage-N1/stage-IIA OBC took nearly two thirds of the whole population (n  =  721, 60.60%). Breast-conserving surgery (BCS) were carried out in 120 patients (10.10%) whereas mastectomy was carried out in 422 patients (35.50%). More than half of all patients received radiotherapy and nearly 80% the whole cohort took chemotherapy. For subtypes of OBC, Luminal A subtype was the commonest one (n  =  326, 27.40%) while HER-2 positive OBC merely made up of about 6.20% (n  =  74) (Table 1).

**Table 1:** Demographic and tumor characteristics

Characteristic	Number of patients	Percentage (%)
N	1189	100
Age (year)		
< 40	54	4.54
40–49	176	14.80
50–59	348	29.27
60–69	343	28.85
≥ 70	268	22.54
Mean of age (year)	59.88	
Median of age (year, range)	60.00 (26–85)	
Race		
White	942	79.23
Black	139	11.69
Asian or Pacific Islander	95	7.99
American Indian/Alaska Native	9	0.76
Unknown/other	4	0.34
Marital status		
Not married	449	37.76
Married	699	58.79
Unknown	41	3.45
Grade		
I/II	65	5.47
III	223	18.76
IV	10	0.84
Unknown	891	74.94
Laterality		
Right	565	47.52
Left	624	52.48
Histology		
Ductal carcinoma	476	40.03
Lobular carcinoma	54	4.54
Mixed type	25	2.10
Other	634	53.32
N		
N1mi	31	2.61
N1	721	60.64
N2	228	19.18
N3	209	17.58
No. of examined LN		
1–3	182	15.31
4–9	177	14.89
≥ 10	675	56.77
Other/unknown	155	13.04
No. of positive LN		
1–3	615	51.72
4–9	212	17.83
≥ 10	148	12.45
Other/unknown	214	18.00
Stage		
IB	31	2.61
IIA	722	60.72
IIIA	228	19.18
IIIC	208	17.49
Surgery		
BCS	120	10.09
Mastectomy	422	35.49
None/unknown	647	54.42
Radiation		
Yes	688	57.86
None/unknown	501	42.13
Chemotherapy		
Yes	929	78.13
No/unknown	260	21.87
Type of systemic therapy		
Neoadjuvant	118	9.92
Adjuvant	583	49.03
Neoadjuvant + adjuvant	104	8.75
Only systemic therapy, no surgery	28	2.35
Other	190	15.98
No/unknown	166	13.96
Subtypes		
Luminal A	326	27.42
Luminal B	111	9.34
HER2 positive	74	6.22
Triple negative	148	12.45
Unknown	530	44.58
ER		
Negative	414	34.82
Borderline/unknown	100	8.41
Positive	675	56.77
PR		
Negative	598	50.29
Borderline/unknown	126	10.60
Positive	465	39.11
HER2		
Negative	474	39.87
Positive	187	15.73
Unknown	528	44.41
Vital status		
Alive	917	77.12
Dead	272	22.88

*BCS* breast-conserving surgery, *MRM* modified radical mastectomy, *RM* radical mastectomy

### Comparison of clinicopathological features in different treatment

We extracted OBC patients received either BCT/non-mastectomy [BCS of the breast (n = 120) or no surgical treatment of the breast (n = 628)] or mastectomy into further analysis (Table 2). Totally, 748 women had BCT therapy and 422 patients received mastectomy procedure. No significant difference was in demographic characteristics such as racial composition (*P* = 0.14) and marital status (*P* = 0.64). Average age of patients in BCT groups was significantly higher than that in mastectomy group (61.16±11.35 vs. 57.65±11.72, *P* <  0.001). Speaking of features of OBC, no significant difference was found for tumor grade, laterality between BCT and mastectomy group (*P* = 0.30 and *P* = 0.15, respectively). For tumor histology, it was marginally associated with surgical types (*P* = 0.04). For lymph node stage, majority of patients in BCT group had a relatively early N1 stage (64.30%) whereas proportion of patients in mastectomy group with stages of N2 and N3 were higher than that of patients in BCT group (22.51% vs. 17.51% for N2; 19.43% vs. 16.58% for N3, *P* <  0.001). A larger proportion of patients in mastectomy group were examined more than 10 lymph nodes (65.40% vs. 52.14%, *P* <  0.001) while a smaller part of patients in mastectomy group were examined 1-3 lymph nodes (11.61 vs. 17.51, *P* <  0.001). For number of positive lymph nodes, about half of the patients in both groups had 1–3 positive nodes but a larger proportion of patients in mastectomy group got 4–9 positive nodes (22.04% vs. 15.64%, *P* <  0.001). More than 60% of the patients in BCT group were diagnosed as IIA (64.30% vs. 53.55%) OBC while a higher proportion of population in mastectomy group were diagnosed as higher stages of OBC (IIIA: 22.75% vs. 17.51%; IIIC 19.19% vs. 16.58%, *P* <  0.001). Rate of Luminal A OBC topped in both BCS groups and mastectomy groups, with 28.61% and 25.12% respectively. HER-2 positive OBC has the lowest rate in both groups (less than 10%). When discussing three immunohistochemistry results apart (ER/PR/HER-2), however, no significant association was found between each of them with surgical procedure (*P*>0.05). Regarding non-surgical management, higher proportion of patients in BCT received radiation compared with that of patients in mastectomy group (61.76% vs. 50.95%, P <  0.001). Different from radiotherapy, a greater percentage of patients in mastectomy group received chemotherapy than that of patients in BCT group (83.18% vs. 75.67%, *P* = 0.003). For patterns of systemic therapy, sequential neoadjuvant and adjuvant therapy were more popular in population from mastectomy group compared with that from BCT group (12.80% vs. 6.55%, *P* <  0.001).

**Table 2:** Basic data of variables in OBC patients with breast-conserving therapy (BCT) and mastectomy

Variables	Surgery				*P*-value
	BCT		Mastectomy		
	N	Percentage (%)	N	Percentage (%)	
N	748	100	422	100	
Age					< 0.001
< 40	24	3.21	29	6.87	
40–49	91	12.17	83	19.67	
50–59	216	28.88	126	29.86	
60–69	223	29.81	114	27.01	
≥ 70	194	25.94	70	16.59	
Mean (± SD)	61.16 ± 11.35		57.65 ± 11.72		< 0.001*
Race					0.14**
White	587	78.48	340	80.57	
Black	92	12.30	45	10.66	
Asian or Pacific Islander	57	7.62	36	8.53	
American Indian/Alaska Native	9	1.20	0	0	
Unknown/other	3	0.40	1	0.24	
Marital status					0.64
Not married	289	34.17	153	36.3	
Married	433	61.67	256	60.7	
Unknown	26	4.17	13	3.1	
Grade					0.30
I/II	35	4.68	28	6.64	
III	135	18.05	85	20.14	
IV	7	0.94	2	0.47	
Unknown	571	76.34	307	72.75	
Laterality					0.15
Right	342	45.72	212	50.24	
Left	406	54.28	210	49.76	
Histology					0.04
Ductal carcinoma	277	37.03	192	45.50	
Lobular carcinoma	37	4.95	16	3.79	
Mixed type	16	2.14	9	2.13	
Other	418	55.88	205	48.58	
N					< 0.001
N1mi	12	1.60	19	4.50	
N1	481	64.30	226	53.55	
N2	131	17.51	95	22.51	
N3	124	16.58	82	19.43	
No. of examined LN					< 0.001
1–3	131	17.51	49	11.61	
4–9	100	13.37	72	17.06	
≥ 10	390	52.14	276	65.40	
Other/Unknown	127	16.98	25	5.92	
No. of positive LN					< 0.001
1–3	376	50.27	227	53.79	
4–9	117	15.64	93	22.04	
≥ 10	86	11.50	60	14.22	
Other/Unknown	169	22.59	42	9.95	
Stage					< 0.001
IB	12	1.60	19	4.50	
IIA	481	64.30	226	53.55	
IIIA	131	17.51	96	22.75	
IIIC	124	16.58	81	19.19	
Radiation					< 0.001
Yes	462	61.76	215	50.95	
None/Unknown	286	38.24	207	49.05	
Chemotherapy					0.003
Yes	566	75.67	351	83.18	
No/Unknown	182	24.33	71	16.82	
Type of systemic therapy					< 0.001
Neoadjuvant	61	8.16	55	13.03	
Adjuvant	380	50.80	196	46.45	
Neoadjuvant + adjuvant	49	6.55	54	12.80	
Only systemic therapy, no surgery	28	3.74	0	0	
Other	113	15.11	74	17.54	
No/Unknown	117	15.64	43	10.19	
Subtypes					0.48
Luminal A	214	28.61	106	25.12	
Luminal B	65	8.69	44	10.43	
HER2 +	51	6.82	23	5.45	
Triple negative	93	12.43	52	12.32	
Unknown	325	43.45	197	46.68	
ER					0.89
Negative	259	34.63	149	35.31	
Borderline/Unknown	61	8.16	37	8.77	
Positive	428	57.22	236	55.92	
PR					0.76
Negative	372	49.73	215	50.95	
Borderline/Unknown	77	10.29	47	11.14	
Positive	299	39.97	160	37.91	
HER2					0.44
Negative	307	41.04	158	37.44	
Positive	118	15.78	67	15.88	
Unknown	323	43.18	197	46.68	

*BCT* breast-conserving therapy, *ER* estrogen, *PR* progesterone receptor, *HER-2* human epidermal growth factor receptor-2, *SD* standard deviation

^*^Wilcoxon Rank Sum and Signed Rank Tests

^**^Fisher's Exact Test

### Survival analysis in OBC cohort

**Fig. 1:**
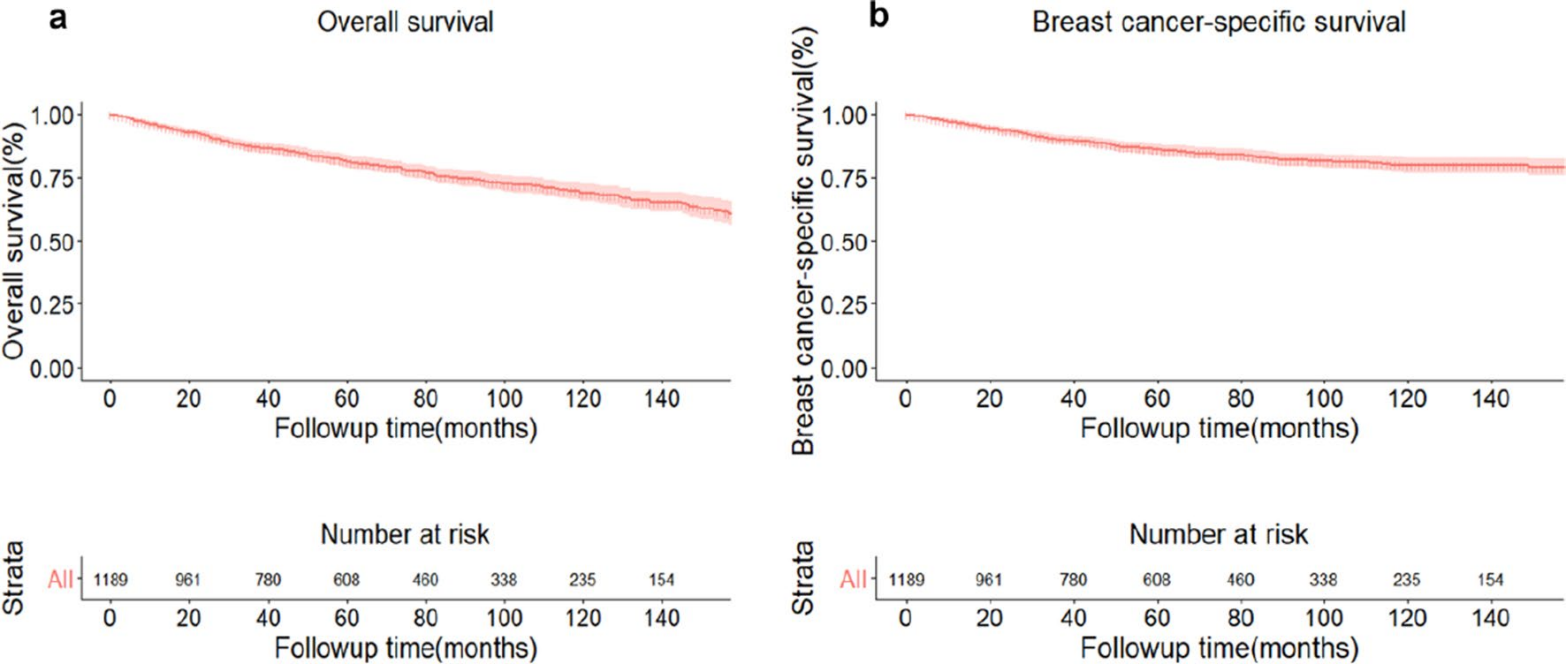
Curves of overall survival (**a**) and breast cancer-specific survival (**b**) of the whole cohort of occult breast cancer patients

**Fig. 2:**
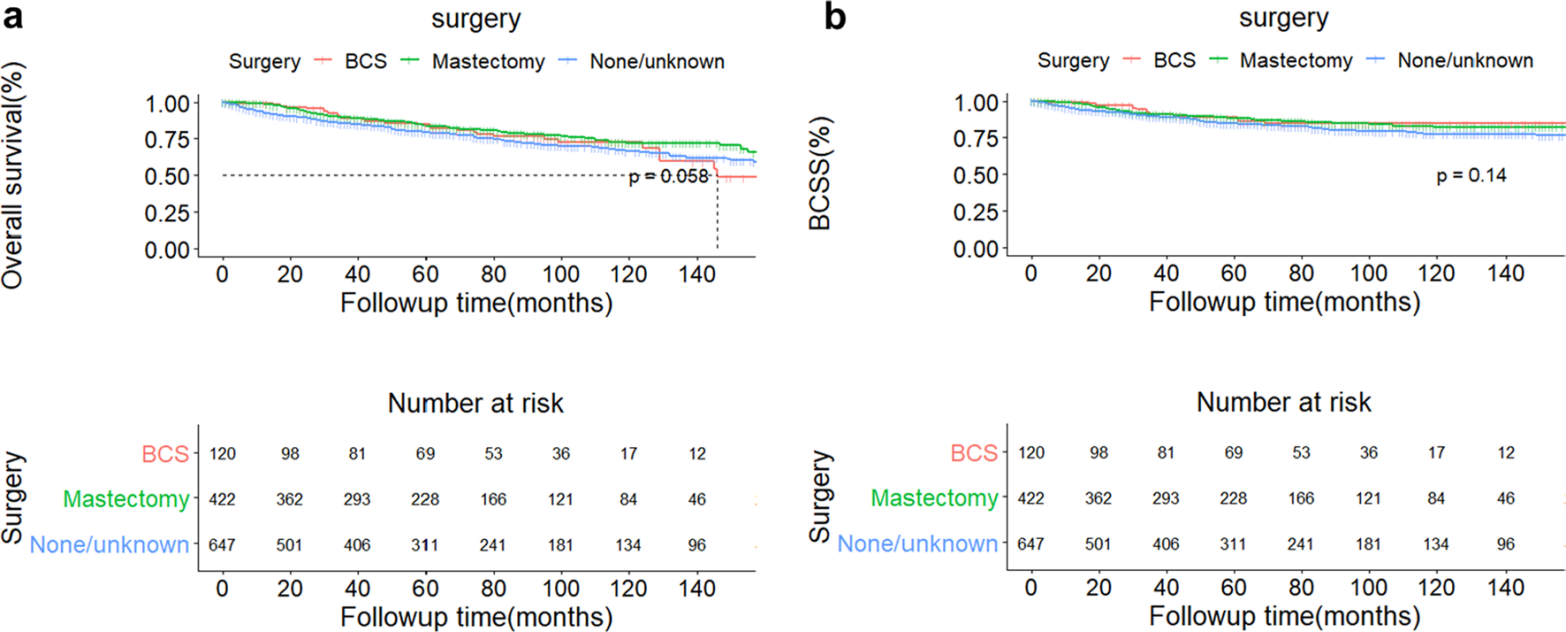
Curves of overall survival (**a**) and breast cancer-specific survival (**b**) of 3 cohorts of patients receiving BCS (pink), mastectomy (green) and no surgery (blue). The dotted line in (**a**) marked the median overall survival timepoint of patients receiving BCS. *BCS* breast-conserving surgery

In the whole OBC cohort, the median follow-up duration was 62 months. Totally, 272 people died from all reasons. 168 died because of disease of breast cancer, among which 14 patients received BCS and 53 female received mastectomy. The 5-year and 10-year overall survival of the whole population was 81.60% (95% CI:79.20–84.10%) and 68.8% (95%CI: 65.30–72.40%), respectively (Fig. 1a). The 5-year and 10-year OBC-specific survival of the whole population was 86.10% (95% CI:83.90–88.30%) and 79.7% (95%CI: 76.80–82.80%), respectively (Fig. 1b). No matter OS and BCSS for the whole cohort, both of them failed to reach median survival timepoints. And both patients in BCS group and mastectomy group were followed up for a median time of 62 months. For population in BCS group, the 5-year and 10-year OS was 84.50% (95% CI 77.60–92.10%) and 72.60% (95% CI: 63.30–83.30%) (Fig. 2a); the 5-year and 10-year BCSS was 88.50% (95% CI 82.30–95.20%) and 84.40% (95% CI: 71.10–92.40%) (Fig. 2b). For mastectomy cohort, the 5-year and 10-year OS was 83.50% (95% CI 79.60–87.60%) and 71.90% (95% CI: 66.20–78.00%) (Fig. 2a); the 5-year and 10-year BCSS was 87.80% (95% CI 84.40–91.30%) and 82.00% (95% CI: 77.30–87.00%) (Fig. 2b). The median survival time of OS for BCS group was 12.17 months while median survival time for mastectomy group and median survival time of BCSS for BCS group were not reached (Fig. 2a, b).

**Fig. 3:**
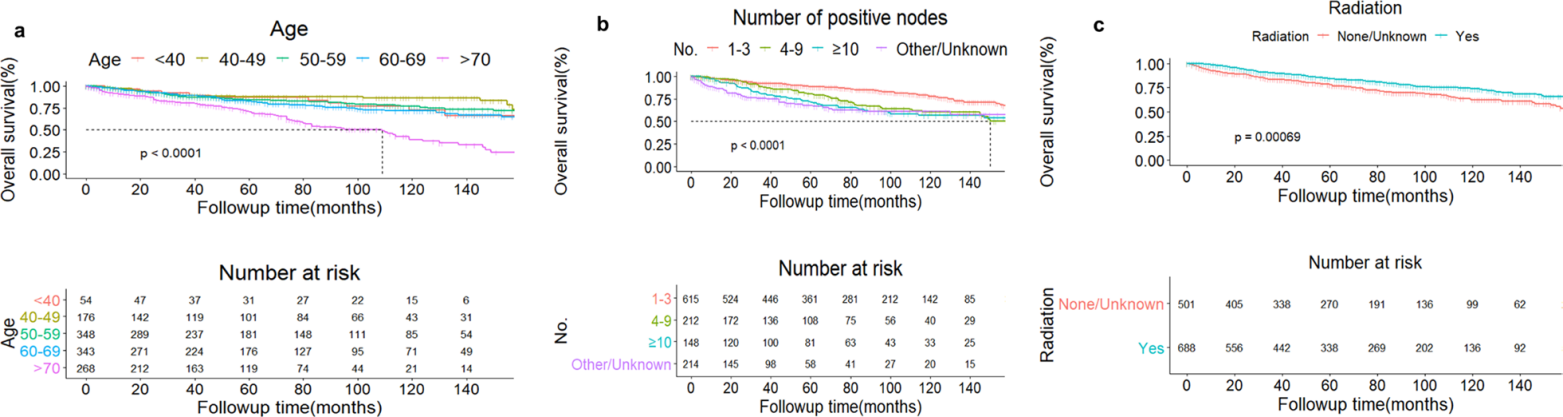
Curves of overall survival according to 5 groups based on different ages (**a**), 4 groups based on different number of positive nodes (**b**) and 2 groups based on different radiation status (**c**). The dotted line in Fig. 3A marked the median overall survival timepoint of patients older than 70 (**a**) and patients with 4–9 positive nodes (**b**)

**Fig. 4:**
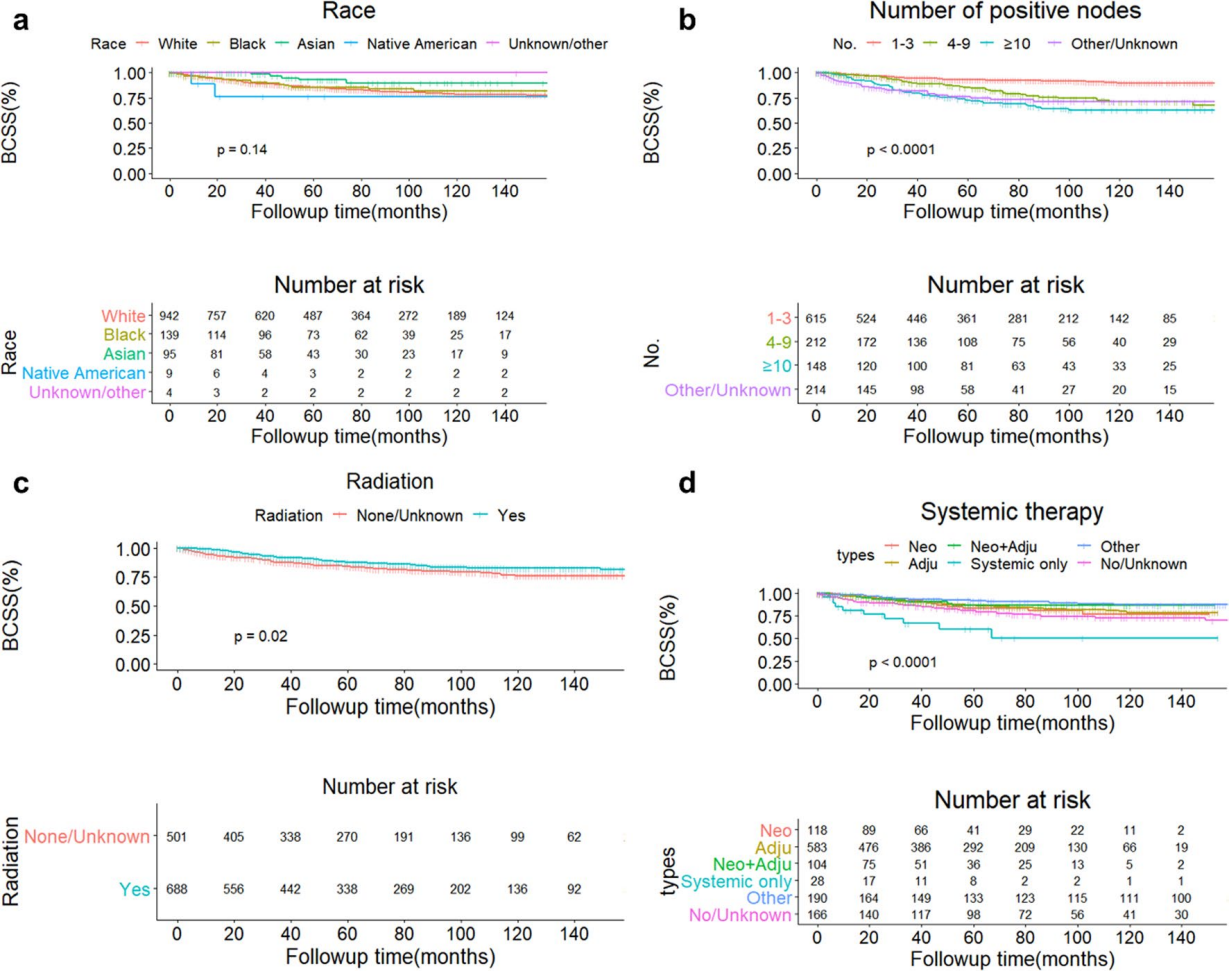
Curves of BCSS according to 5 groups based on different races (**a**), 4 groups based on different number of positive nodes (**b**), 2 groups based on different radiation status (**c**) and 6 groups based on different patterns of systemic therapy (**d**). *Adju* adjuvant systemic therapy, *BCSS* breast cancer-specific survival, *Neo* neoadjuvant systemic therapy

Univariate and multivariate COX regression analysis for OS and BCSS were conducted (Tables 3 and 4, respectively). Age, marital status, grade, N staging, number of positive lymph node, stage, radiation, chemotherapy, type of systemic therapy, tumor subtypes, ER and PR were factors significantly associated with OS according to univariate analysis (*P* <  0.10) (Table 3). By multivariate analysis, it was found that age, number of positive lymph nodes and radiation were independent predictive factors for OS (*P* <  0.05) (Table 4; Fig. 3a–c). For BCSS, age, race, grade, N staging, number of positive lymph node, stage, radiation, chemotherapy, type of systemic therapy, tumor subtypes, ER and PR were selected to enter multivariate analysis (*P* <  0.10) (Table 3). In multivariate analysis, independent predictive factors of BCSS included race, number of positive lymph node, radiation and types of systemic therapy (*P* <  0.05) (Table 4; Fig.4a–d). Patients older than 70 has a significant gloomy overall survival than that with age less than 40 (hazard ratio, HR = 2.15, 95% CI = 1.13–4.08). Compared with white OBC patients, Asian/Pacific-island patients tended to get a better BCSS (HR = 0.39, 95%CI = 0.16–0.97, *P* = 0.04) but native Americans had a relatively poor survival (HR = 4.25, 95% CI = 1.01–17.99, *P* = 0.04). More positive lymph node is associated with both poor OS and BCSS (*P* < 0.001). Patients receiving radiation tended to get a better prognosis no matter statistical analysis of OS or BCSS (Table 4).

**Table 3:** Univariable analysis of OS and BCSS in OBC patients

Variables	OS			BCSS		
	HR	95% CI	*P*-value	HR	95% CI	*P*-value
Age (year)	1.05	1.03–1.06	< 0.0001	1.03	1.02–1.04	< 0.001
< 40	Reference			Reference		
40–49	0.63	0.31–1.31	0.22	0.82	0.34–1.97	0.66
50–59	0.93	0.49–1.77	0.83	0.99	0.44–2.20	0.98
60–69	1.14	0.60–2.16	0.68	1.10	0.50–2.45	0.81
≥ 70	2.69	1.44–5.01	0.002	2.03	0.93–4.46	0.08
Race						
White	Reference			Reference		
Black	1.30	0.93–1.81	0.12	0.88	0.55–1.42	0.61
Asian or Pacific Islander	0.75	0.44–1.26	0.28	0.37	0.15–0.89	0.03
American Indian/Alaska Native	1.11	0.28–4.49	0.88	1.78	0.44–7.17	0.42
Unknown/other	8.34 × 10^–7^	0-INF	0.99	7.65 × 10^−7^	0-INF	0.99
Marital status						
Not married	Reference			Reference		
Married	0.71	0.56–0.91	0.007	0.77	0.56–1.06	0.11
Unknown	1.25	0.69–2.26	0.47	1.31	0.63–2.72	0.47
Grade						
I/II	Reference			Reference		
III	2.59	1.03–6.54	0.04	2.59	1.03–6.54	0.04
IV	0.99	0.12–8.52	1.00	0.99	0.12–8.52	1.00
Unknown	1.81	0.74–4.42	0.20	1.81	0.74–4.42	0.20
Laterality						
Right	Reference			Reference		
Left	1.03	0.81–1.31	0.79	1.11	0.82–1.51	0.50
Histology						
Ductal carcinoma	Reference			Reference		
Lobular carcinoma	0.85	0.44–1.62	0.61	1.24	0.59–2.60	0.57
Mixed type	0.69	0.28–1.70	0.43	0.77	0.24–2.47	0.67
Other	0.85	0.66–1.08	0.18	1.03	0.75–1.43	0.84
N						
N1mi	Reference			Reference		
N1	2.15	0.53–8.70	0.28	1.01	0.32–16.46	0.41
N2	2.84	0.69–11.67	0.15	1.01	0.64–33.65	0.13
N3	4.00	0.98–16.32	0.05	1.01	1.04–54.08	0.05
No. of positive LN						
1–3	Reference			Reference		
4–9	1.82	1.32–2.52	< 0.001	2.77	1.79–4.28	< 0.001
≥ 10	2.22	1.59–3.10	< 0.001	4.42	2.91–6.71	< 0.001
Other/Unknown	1.01	1.99–3.77	< 0.001	4.09	2.67–6.25	< 0.001
Stage						
IB	Reference			Reference		
IIA	2.15	0.53–8.70	0.28	1.01	0.32–16.46	0.4123
IIIA	2.84	0.69–11.63	0.15	1.01	0.63–33.51	0.1312
IIIC	4.02	0.98–16.40	0.05	1.01	1.04–54.37	0.0453
Surgery						
BCS	Reference			Reference		
Mastectomy	0.82	0.53–1.26	0.36	1.08	0.60–1.94	0.80
None/Unknown	1.13	0.76–1.69	0.55	1.44	0.82–2.52	0.20
Radiation						
None/Unknown	Reference			Reference		
Yes	0.66	0.52–0.84	0.0007	0.70	0.52–0.95	0.02
Chemotherapy						
Yes	Reference			Reference		
No/Unknown	0.55	0.43–0.71	< 0.001	0.73	0.52–1.02	0.07
Type of systemic therapy						
Neoadjuvant	Reference			Reference		
Adjuvant	0.97	0.61–1.55	0.91	0.92	0.53–1.60	0.76
Neoadjuvant + adjuvant	0.53	0.24–1.15	0.11	0.73	0.32–1.66	0.45
Only systemic therapy, no surgery	3.42	1.71–6.83	0.0005	3.58	1.61–7.97	0.002
Other	0.27	0.37–1.08	0.09	0.52	0.27–1.04	0.06
No/Unknown	0.25	1.01–2.70	0.04	1.39	0.76–2.53	0.28
Subtypes						
Luminal A	Reference			Reference		
Luminal B	0.62	0.30–1.27	0.19	0.69	0.28–1.66	0.40
HER2 positive	1.31	0.70–2.43	0.40	1.80	0.89–3.64	0.10
Triple negative	1.63	1.03–2.57	0.04	2.03	1.17–3.54	0.01
Unknown	1.28	0.91–1.81	0.16	1.52	0.98–2.35	0.06
ER						
Negative	Reference			Reference		
Borderline/Unknown	1.08	0.73–1.61	0.70	1.18	0.73–1.90	0.50
Positive	0.71	0.55–0.92	0.009	0.59	0.43–0.81	0.001
PR						
Negative	Reference			Reference		
Borderline/Unknown	1.08	0.76 -1.56	0.66	1.06	0.68–1.66	0.80
Positive	0.78	0.60–1.01	0.06	0.54	0.38–0.77	0.0007
HER2						
Negative	Reference			Reference		
Positive	0.74	0.46–1.19	0.21	0.85	0.49–1.47	0.56
Unknown	1.08	0.81–1.43	0.62	1.15	0.81–1.63	0.43

*CI* confidence interval, *ER* estrogen receptor, *HER-2* human epidermal growth factor receptor-2, *N* N staging of OBC, *PR* progesterone receptor

**Table 4:** Multivariable analysis of OS and BCSS in OBC patients

Variables	OS			BCSS		
	HR	95% CI	*P*-value	HR	95% CI	*P*-value
Age (year)						
< 40	Reference					
40–49	0.57	0.27–1.19	0.13	0.64	0.26–1.56	0.32
50–59	0.82	0.43–1.58	0.56	0.82	0.36–1.85	0.64
60–69	1.04	0.55–1.99	0.90	0.97	0.43–2.18	0.93
≥ 70	2.15	1.13–4.08	0.02	1.52	0.67–3.41	0.31
Race						
White	Reference			Reference		
Black	–	–	–	0.90	0.56–1.47	0.68
Asian or Pacific Islander	–	–	–	0.39	0.16–0.97	0.04
American Indian/Alaska Native	–	–	–	4.25	1.01–17.99	0.05
Unknown/other	–	–	–	1.23 × 10^–6^	0-INF	0.99
Marital status						
Not married	Reference			–	–	–
Married	0.80	0.62–1.02	0.07	–	–	–
Unknown	1.25	0.68–2.31	0.48	–	–	–
Grade						
I/II	Reference			Reference		
III	0.90	0.51–1.59	0.71	1.71	0.65–4.47	0.27
IV	0.66	0.22–2.04	0.47	0.65	0.07–5.73	0.70
Unknown	0.70	0.41–1.19	0.18	1.26	0.50–3.16	0.63
N						
N1mi	Reference			Reference		
N1	1.34 × 10^11^	0-INF	0.99	1.17 × 10^13^	0-INF	1.00
N2	1.72 × 10^6^	0-INF	0.99	4.04 × 10^7^	0-INF	1.00
N3	3.44	0.79–14.92	0.10	4.47	0.58–34.38	0.15
No. of positive LN						
1–3	Reference			Reference		
4–9	2.09	1.17–3.73	0.01	1.96	0.95–4.02	0.07
≥ 10	1.53	0.86–2.73	0.15	2.28	1.16–4.46	0.02
Other/Unknown	2.47	1.76–3.47	< 0.001	3.26	2.07–5.13	< 0.001
Stage						
IB	Reference			Reference		
IIA	1.39 × 10^–11^	0-INF	0.99	1.45 × 10^–13^	0-INF	1.00
IIIA	9.80 × 10^–7^	0-INF	0.99	6.46 × 10^–8^	0-INF	1.00
IIIC	NA	NA	NA	NA	NA	NA
Radiation						
None/Unknown	Reference			Reference		
Yes	0.68	0.52–0.88	0.003	0.63	0.45–0.88	0.007
Chemotherapy						
Yes	Reference			Reference		
No/Unknown	0.87	0.52–1.44	0.59	1.08	0.51–2.28	0.84
Type of systemic therapy						
Neoadjuvant	Reference			Reference		
Adjuvant	0.93	0.58–1.50	0.77	1.00	0.57–1.75	0.99
Neoadjuvant + adjuvant	0.50	0.23–1.10	0.08	0.69	0.30–1.59	0.38
Only systemic therapy, no surgery	1.88	0.90–3.90	0.09	2.40	1.03–5.60	0.04
Other	0.55	0.32–0.96	0.04	0.46	0.23–0.93	0.03
No/Unknown	1.02	0.51–2.06	0.95	1.23	0.48–3.17	0.67
Subtypes						
Luminal A	Reference			Reference		
Luminal B	0.63	0.31–1.31	0.22	0.65	0.26–1.60	0.35
HER2 positive	1.10	0.54–2.22	0.79	1.31	0.56–3.02	0.53
Triple negative	1.42	0.81–2.50	0.22	1.66	0.81–3.40	0.17
Unknown	1.53	1.02–2.27	0.04	1.64	0.97–2.76	0.06
ER						
Negative	Reference			Reference		
Borderline/Unknown	1.13	0.51–2.54	0.76	1.63	0.63- 4.18	0.31
Positive	0.78	0.53–1.14	0.20	0.87	0.52–1.43	0.57
PR						
Negative	Reference			Reference		
Borderline/Unknown	0.85	0.41–1.79	0.68	0.72	0.29–1.74	0.46
Positive	0.99	0.69–1.43	0.98	0.78	0.49–1.24	0.30

*BCSS* breast cancer-specific survival, *CI* confidence interval, *ER* estrogen receptor, *HR* hazard ratio, *INF* infinite, *N* N staging of OBC, *NA* not available, *OS* overall survival, *PR* progesterone receptor

## Discussion

In our study, we collected nearly 1200 OBC patients for 15 years and the data is the most up-to-date one. We found that OBC was predominated by elderly population. Majority of OBC patients were diagnosed at relative early stage (Stage IIA) and Luminal A type of OBC took up nearly 30% of all subjects. Radiotherapy and chemotherapy were common and important treatments for OBC patients. In this study, we found that older patients tended to receive less-invasive surgical intervention. Patients in mastectomy group were found more involved lymph nodes thus more patients were diagnosed as advanced stage OBC (stage IIIA and IIIC) compared with subjects in BCT group. Patients treated by radiotherapy had a significant difference between two surgical groups, in which more than 60% subjects in BCT group were accepted this therapy but only half of women took radiotherapy in mastectomy groups (*P* <  0.001). Speaking of survival analysis, the whole OBC cohort generally had optimistic prognosis. 5-year OS and BCSS exceeding 80% and both of the survival analysis did not reach the timepoint of median survival. When the whole cohort was grouped according to types of surgery, both of the 5 and 10-year BCSS in BCS and mastectomy group were higher than 5 and 10-year OS in the same group. However, no significant difference was found no matter in OS or BCSS between BCS and mastectomy group (*P* = 0.058 for OS and *P* = 0.14 for BCSS). For Cox regression analysis, we found that age was an independent predictor for OS and higher age meant a poorer survival. Race served as an independent predictor for BCSS and Asian/Pacific natives has a better clinical outcome while native Americans had a relatively poor survival. Radiotherapy was clinically beneficial to OBC patients, no matter according to OS or BCSS. But no significant benefit was found between survival and systemic therapy. This finding was also supported by the result derived from multivariate analysis of tumor subtypes immunohistochemistry: ER, PR, HER-2 and tumor subtypes were not independent predictors for neither OS or BCSS.

Although NCCN guidelines stated that mastectomy was recommended for OBC patients, the optimal surgical therapy for the ipsilateral breast in OBC has been controversial [14]. According to a meta-analysis collecting 7 qualified studies, no significant difference in overall survival between ALND plus mastectomy and ALND combined with breast radiation [7]. Likewise, in a clinical trial carried on by Rueth’s team, no significant survival disparity was found between BCT and mastectomy (*P* = 0.7). No local and distal recurrence occurred within 5 years, either [15]. A clinical study based on National Cancer Database collected 190 patients diagnosed from 2004 to 2014 [16]. Treatment strategies included mastectomy alone, radiation alone and mastectomy combined with radiation. No significant difference in OS was found between each two of these three strategies (mastectomy vs. radiation, *P* = 0.650; mastectomy + radiation vs. mastectomy, *P* = 0.393; mastectomy vs. radiation, *P* = 0.872). These findings are also supported by two retrospective study from China and Korean respectively, suggesting that mastectomy was not more clinically beneficial than BCT [8, 17]. Even some studies found that a less-intensive approach would be beneficial for OBC patient. In another research also based on National Cancer Database, 1231 OBC patients were grouped into MRM± radiotherapy (N = 592), radiotherapy + ALND (N = 342), ALND alone (N = 106) and no breast surgery (N = 191). They found that patients treated by ALND and radiotherapy have significantly better OS compared with patients received MRM with/without radiotherapy (HR = 0.475, 95% CI 0.306-0.736, *P*  =  0.001). Multivariate analysis proved that ALND with radiation was an independent protective predictor of OS (HR 0.509, 95% CI 0.321-0.808, *P* =  0.004). However, a retrospective study performed by Wang et al. drew an opposite conclusion. Their study included 51 OBC cases from a single center, in which 38 had ALND plus Mastectomy and the other 13 patients had ALND only [18]. 28 of 38 patients having mastectomy had been found the primary tumor in the breast by pathology. Recurrence rate in mastectomy group was 26%, much lower than that in ALND-only group (77%). Patients with mastectomy had a significantly promising disease-free survival and overall survival compared with patients merely received ALND (*P* <  0.001). Hence, this study drew a conclusion that mastectomy based on ALND is necessary for OBC patients.

For radiotherapy, we found that radiotherapy is clinically beneficial for OBC. This finding is in line with majority of previous clinical research. A study from the UK collected 29 OBC patients across more than 25 years. Median follow-up time was 44 months. Amongst, 16 patients got local radiotherapy and 13 patients did not receive local radiation. Local recurrence rate was 12.50% in radiation group while 69% of patients in non-radiotherapy group got local recurrence (*P* = 0.02). Moreover, radiotherapy could significantly improve relapse-free survival (HR = 0.31, *P* = 0.04) and local relapse-free survival (HR = 0.09, *P* =  0.004). Overall survival, unfortunately, was not positively impacted by radiation therapy [19]. In 2010, another research conducted by team from UK led by Masinghe reported 53 OBC patients diagnosed from 1974 to 2003. Similar to the above studies, this study was also a mixture of clinical and pathological OBC. All the patients received axillary surgery but only 25 of them (47%) had ALND. 5-year recurrence rate of ipsilateral breast was 16.40% (95% CI 4.30–28.50%) in population receiving radiotherapy, lower than that in non-radiation population (35.80%, 95% confidence interval 7.60–64.10%). Similar results were presented in 10-year ipsilateral relapse rate, where radiotherapy group was 23.20% (95% CI 8.90–37.60%) and non-radiotherapy group was 51.90% (95% CI 17.40–86.40%). Furthermore, patients receiving radiotherapy had a significantly higher 5-year and 10-year BCSS rate compared with patients without radiation [*P* = 0.0073; 5-year: 72.80% (95% CI 59.10–86.50%) vs. 58.30% (95% CI 30.40–86.20%); 10-year: 66.20% (95% CI 51.00–81.50%) vs. 14.6% (0.00–43.30%), respectively). Radiotherapy was the only significant predictor of survival [20]. In a Korean study, 3 of 66 OBC patients did not receive breast radiation. In 15 patients received BCS therapy (blind local excision of the breast), no positive finding of tumor was identified in all these patients. The local recurrence rate was significantly lower in patients without breast radiation than that in patients with breast radiation (6.30% vs. 66.70%; *P*  =  0.02). Breast radiation could significantly improve 8-year disease-free survival in patients with breast radiation compared with patients without ipsilateral breast radiation (89.50% vs. 50.00%, *P* = 0.02). The shortcoming of this study was that number of patients without breast radiation was much lower than that of radiation group so that the conclusion should be further examined [21]. Different from studies above, research team from Memorial Sloan Kettering Cancer Center gave an opposite answer for this topic. In this study, all the 38 OBC patients were evaluated by MRI before recruited, stricter examination than the above studies. Among the subjects, 25 patients received ALND + whole breast radiotherapy while 13 patients got MRM (6 patients got chest-wall radiation) [22]. This totally met the criteria of NCCN guideline mentioned before. To make the population in different groups more homogenous, all the patients got chemotherapy. Surprisingly, no recurrence occurred in lymph node during a median follow-up of 7 years. Even relapse of two breast occurred in patients receiving ALND combined with whole breast radiotherapy. No local recurrence occurred in patients with MRM. Similar rates of distal failure were between the two groups (7.70% in MRM cohort vs. 8.00% in ALND + radiotherapy group). Nevertheless, the conclusion of this study should be further discussed because of a small sample size.

For systemic therapy, especially chemotherapy for OBC patients, no specific guidelines, recommendation and consensus have been laid out. Conventionally, neoadjuvant chemotherapy was commonly used for locally advanced/ inoperable/inflammatory breast cancers [23]. This kind of regimen can degrade the tumor stage and serve to evaluate treatment response [24]. Several studies reported results of effectiveness of neoadjuvant chemotherapy in treating of OBC. A case series from China including 5 OBC patients suggested an effective neoadjuvant chemotherapy with pathological complete response (PCR) rate reached 80% (4/5). Among the 4 patients, they were all received mastectomy followed by radiotherapy, two of whom also underwent endocrine therapy. Another study led by Rueth et.al collected 36 OBC patients. Nearly 95% of subjects (34/36) received chemotherapy and the rate of neoadjuvant therapy was up to almost 70% (25/36). In 33 patients with ALND, 15 of them got neoadjuvant therapy. Thus, the PCR rate of neoadjuvant therapy was about 60%, which supported the effectiveness of neoadjuvant therapy in the case series above [15]. In the most recent study with the largest sample size, Cohen et.al collected 684 OBC patients and 214 (31.3%) of them received neoadjuvant chemotherapy. PCR was recognized in 55 of 214 (26%) patients took neoadjuvant therapy. In our study, counterintuitively, we found that patients without chemotherapy tended to have a better prognosis, partly because patients administered with chemotherapy had a relatively higher tumor stage. In our study, 21% cases with stage-IIIA OBC and 19% cases with stage-IIIC cases in chemotherapy group patients, whereas both of the stage-IIIA and stage-IIIC OBC patients accounted for 13% in the other group (*P* = 0.002, χ^2^test). As the number of studies focusing on neoadjuvant chemotherapy was small and the PCR rate varied among these studies by virtue of sample size in huge disparity, it is still debatable whether systemic therapy is beneficial for OBC patients.

In our study, several limitations are necessary to be noticed: (1) For systemic therapy, current database is shortage of specific data such as the chemotherapy formula and dosage of each drug. Thus, we could not get more information about chemotherapy and its relationship with survival. Moreover, data of endocrine therapy is not available in current version of SEER database; (2) SEER database predominately describes clinical characteristic of American citizens including white and black citizens. Data of Asian OBC patients were insufficient so that it is unclear whether the results of this analysis were also generalized to Asian population; (3) Because the data of HER-2 status originates from 2010, it is not available in the cases diagnosed before 2010, which impacted the descriptive results and following analysis about HER-2 status, tumor subtypes as well as their associations with OS and BCSS; (4) For radiation part in SEER database, we did not know the exact number of patients unknown information of radiation and we could not separately summarize number of patients with or without radiation. Hence, some sort of deviation of the survival result might be existed. We hope that more clear information can be retrieved in updated edition of this database.

## Conclusion

Majority of OBC patients had a relatively optimistic prognosis. A less-intensive surgical therapy is acceptable for OBC treatment without cost on OS or BCSS. Radiotherapy was beneficial for clinical outcomes of OBC patients. More information of systemic therapy is expected to be obtained to refine the treatment recommendation for OBC.

## Data Availability

The datasets generated and integrated are available in Surveillance, Epidemiology, and End Results (SEER) database (https://seer.cancer.gov/). We selected the SEER research plus data, 18 Registries, Nov 2020 Sub (2000–2018) to perform further analysis. Software for data retrieving and integration is SEER* Stat 8.3.9.1, which is successfully downloaded from official website of SEER database (https://seer.cancer.gov/seerstat/).

## Abbreviations

ALND: Axillary lymph node dissection
BCS: Breast-conserving surgery
BCT: Breast-conserving therapy
BCSS: Breast-cancer specific survival
CI: Confidence intervals
ER: Estrogen receptor
HER-2: Human epidermal factor receptor-2
HR: Hazard ratio
MRI: Magnetic resonance imaging
MRM: Modified radical mastectomy
NCCN: National Comprehensive Cancer Network
OBC: Occult breast cancer
OS: Overall survival
PCR: Pathological complete response
PR: Progesterone receptor
SEER: Surveillance, Epidemiology, and End Results
